# Reversal of Taxol resistance in hepatoma by cyclosporin A: involvement of the PI-3 kinase-AKT 1 pathway

**DOI:** 10.1038/sj.bjc.6600788

**Published:** 2003-03-18

**Authors:** H-L Lin, W-Y Lui, T-Y Liu, C-W Chi

**Affiliations:** 1 Institute of Pharmacology, National Yang-Ming University, Taipei, Taiwan; 2 Department of Surgery, Taipei Veterans General Hospital, Taipei, Taiwan; 3 Department of Medical Research and Education, Taipei Veterans General Hospital, Taipei, Taiwan; 4 School of Medicine, National Yang-Ming University, Taipei, Taiwan

**Keywords:** hepatoma, drug resistance, cyclosporin, Taxol

## Abstract

Hepatoma cells are known to be highly resistant to chemotherapy. Previously, we have found differential Taxol resistance in human and murine hepatoma cells. The aim of this study was to examine the effect of a multidrug resistance inhibitor, cyclosporin A in combination with Taxol on hepatoma *in vitro* and *in vivo*, and to identify the possible mechanism involved in Taxol resistance. Simultaneous treatment of cyclosporin A (0–10 *μ*M) and Taxol (0.1 *μ*M) inhibited cell growth *in vitro*. Cyclosporin A interfered with Taxol (0.1 *μ*M)-induced AKT activation and BAD phosphorylation. Cyclosporin A combined with Taxol treatment augments caspase-9, -3 activation and loss of mitochondrial membrane potential in HepG2 cells. PI3 kinase inhibitor, wortmannin, or a dominant-negative AKT1 expression vector treatment partially enhanced Taxol-induced apoptosis indicating that PI3 kinase-AKT pathway was involved in Taxol-resistance pathway. Moreover, combination treatment reduced tumour growth in SCID and C57BL/6 mice as compared to either Taxol or cyclosporin A treatment. Our results indicate that the combination of cyclosporin A and Taxol is effective in the reversal of Taxol resistance through the inhibition of PI3 kinase-AKT1 pathway.

Hepatocellular carcinoma (HCC) is highly resistant to chemotherapy and the curative approach is surgical resection. However, tumour location and number as well as frequent recurrence rate limited the survival of patients with HCC (Nagasue *et al*, 1993; Shimada *et al*, 1996; Nonami *et al*, 1997). Thus, preoperative or postoperative adjuvant chemotherapy could be used to improve the surgical results (Nagasue *et al*, 1993; Izumi *et al*, 1994; Tanaka *et al*, 1999). Hepatocellular carcinoma originates from the liver, which is responsible for the detoxification of exogenous and endogenous chemicals. High level of phase I and phase II enzyme activity as well as membrane transporter system (multidrug resistance, MDR and multidrug resistance-related protein, MRP) were documented in the liver (Cavalieri *et al*, 1997) and hepatoma cells (Kool *et al*, 1997; Lin *et al*, 1999). Moreover, HCC grows as solid spheroid-like architecture leading to less drug uptake. Taken together, the intrinsic factors and microenvironment are two important obstacles for chemotherapeutic treatment of patients with HCC.

Taxol, one of the antimicrotubule chemotherapeutic drugs, has been reported to be effective in the treatment of many tumours including ovarian, breast and lung cancers (Chabner, 1991; Rowinsky and Donehower, 1991; Slichenmyer and Von Hoff, 1991) but not in HCC (Chao *et al*, 1998). The well-established action mechanism of Taxol in the treatment of cancer is based on stabilisation of microtubules in tumour cells and induction of apoptosis. In addition, several signal transduction pathways such as Raf, JNK (Torres and Horwitz, 1998; Wang *et al*, 2000) and specific cell cycle phase (Lin *et al*, 1998) have been indicated to be involved in Taxol-elicited apoptotic responses. Moreover, expression of oncogenes or drug resistance factors as well as decoy receptors induction could account for the low response rate after chemotherapy in patients with HCC (Chao *et al*, 1998; Yamanaka *et al*, 2000), including Taxol treatment.

Taxol is known to be metabolised (Harris *et al*, 1994b) in the liver and inactivated (Harris *et al*, 1994a) by cytochrome *P*450 3A4. Cyclosporin A is also metabolised by cytochrome *P*450 3A in the liver (Villeneuve *et al*, 2000) and has been found to block the function of MDR (Twentyman *et al*, 1987). A recent report further showed that cyclosporin A modulated the absorption of oral administration of Taxol and increased the plasma concentration of Taxol in patients with solid tumours (Meerum Terwogt *et al*, 1999). It was suggested that cyclosporin A modulated the function of MDR that led to increased Taxol absorption in the gastrointestinal tract. Whether a combination of cyclosporin A and Taxol will induce Taxol-resistant hepatoma cells to respond to Taxol is worth investigating.

In a previous study, we have found that HepG2 and HA22T/VGH hepatoma cells were highly resistant to Taxol treatment, while Hepa 1-6 and Hep3B cells were relatively more sensitive to Taxol (Lin *et al*, 2000). The objective of this study was to investigate whether cyclosporin A enhanced the responses of Taxol-resistant hepatoma cells to Taxol treatment *in vitro* and *in vivo*. We show that reversal of Taxol resistance in hepatoma cells was observed. In addition, we show that the PI3 kinase (phosphoinositide 3-kinase)-AKT1 signalling pathway is involved in Taxol resistance.

## MATERIALS AND METHODS

### Cell culture, transfection and treatment

Human hepatoma cell lines, Hep3B, HepG2, HA22T/VGH, and murine hepatoma cell line, Hepa 1-6, were cultured in DMEM (GIBCO, BRL NY, USA) containing 10% fetal bovine serum (Hyclone, UT, USA), 0. 01 mg ml^−1^ gentamycin and 0.1 mM nonessential amino acid. Cells were grown in a CO_2_ incubator at 37°C, with 5% CO_2_ and 95% filtered air. For transient transfection, HepG2 cells (3 × 10^5^) were cultured in 35-mm dish overnight and transient transfected with pUSEamo(+) vector or dominant-negative AKT1 expression vector (Upstate Biotechnology, NY, USA) by using LIPOFECTAMINE PLUS reagents (GIBCO, BRL NY, USA) according to the manufacturer's instructions. Hepatoma cells were treated with Taxol (Biomol PA, USA) combined with cyclosporin A (Sigma MO, USA) or wortmannin at indicated concentrations. Taxol was dissolved in DMSO as stock solution, the final concentration of DMSO was <0.1% in media. For *in vivo* experiments, Taxol was dissolved in DMSO (5 mg ml^−1^) as stock and diluted with phosphate-buffered saline (PBS) to final concentration.

### Viability assay

Cells were cultured in a 96-well cell culture cluster (COSTAR NY, USA) at a density of 4 × 10^4^ cells ml^−1^. After drug treatment for 24–72 h, the medium was discarded and replaced with an equal volume (100 *μ*l) of fresh medium containing MTT (0.456 mg ml^−1^; 3-[4,5-dimethylthiazol-2-yl]-2,5-diphenyl-tetrazolium bromide) and incubated for 1.5 h at 37°C. The medium was discarded. Cells were then combined with 100 *μ*l DMSO. Cell viability was determined according to the colorimetric comparison by reading OD values from a microplate reader (SPECTRA MAX 250 Molecular Devices, CA, USA) at an absorption wavelength of 570 nm.

### Assay of caspase activity

Caspase activity was determined as described previously (Lin *et al*, 2001). Briefly, cell pellets were collected, washed with PBS and extracted with lysis buffer (20 mM Tris buffer (pH 7.5), 1 mM EDTA, 100 *μ*M phenylmethylsulphonyl fluoride, aprotonin (2 *μ*g ml^−1^), pepstatin (2 *μ*g ml^−1^) and leupeptin (2 *μ*g ml^−1^)). An aliquot of total protein was incubated with reaction buffer (50 mM HEPES, 10% sucrose, 0.1% CHAPS, pH 7.5), dithiothreitol (50.5 mM) and substrates (YVAD-pNA, DEVD-pNA, IETD-pNA or LEHD-pNA for caspase-1, -3, -8 or –9, respectively). Caspase activity was determined according to the colorimetric comparison by reading OD values from a microplate reader at an absorption wavelength of 405 nm. Data are presented as fold of increase as compared to respective control groups.

### Mitochondria membrane potential measurement

Treated cells were harvested and stained with JC-1 (0.2 *μ*g ml^−1^). (Molecular Probe OR, USA) for 10 min in Medium 199 (GIBCO, BRL NY, USA) at 37°C. The loss of mitochondria membrane potential was measured by flow cytometry for the decreased red fluorescence.

### Western blot analysis

Total proteins were extracted with lysis buffer (20 mM Tris buffer (pH 7.5), 1 mM EDTA, 100 *μ*M phenylmethylsulphonyl fluoride, aprotonin (2 *μ*g ml^−1^), pepstatin (2 *μ*g ml^−1^) and leupeptin (2 *μ*g ml^−1^)). Protein quantity was measured by Bradford assay and an equal quantity of total protein (15 *μ*g) was applied on 10 or 12.5% SDS – PAGE. Equal loading of total protein was determined by electrophoresis prior to staining with GelCode® (Pierce IL, USA) for visualisation. After protein transfer onto nitrocellulose membrane, specific protein expression level was measured by using antibodies for AKT1 (Upstate, NY, USA), phosphorylated AKT1 (Upstate, NY, USA), BAD (Santa Cruz CA, USA), phosphorylated BAD (Upstate, NY, USA), PTEN (Santa Cruz CA, USA) and procaspase 3 (IMGENEX CA, USA), respectively, followed by enhanced chemiluminescence detection.

### Flow cytometry

Cells (10 000) were analysed on a Becton Dickinson FACS*Calibur* flow cytometer (San Jose, CA, USA) using an argon-ion laser (15 mW) with an incident beam at 488 nm. For the green or red JC-1 fluorescence, signals were detected by collection through a 530 or 585 nm filter, respectively.

### Animal study

Male SCID mice and C57BL/6 mice with body weight of 20–23 g were obtained from the National Taiwan University and National Laboratory Animal Breeding and Research Center of National Science Council, respectively. SCID mice were maintained in isolated cages with filter bonnet in a room with 12-h dark and light cycle. For experiments, Hepa 1-6 (5 × 10^6^ cells) cells were inoculated s.c. in 7–8-week old SCID or C57BL/6 mice 3 days prior to treatment with cyclosporine A and Taxol. Tumour cells were injected subcutaneously at day 0. After 3 days (day 3), mice with detectable small nodule (0.2 × 0.2 cm^2^ in diameter) were enrolled and then cyclosporin A (5 or 6.67 mg kg^−1^) and/or Taxol (2 or 2.68 mg kg^−1^) administered intraperitoneally at days 3, 4, 9, 10 for SCID mice or days 3, 4, 10, 11, 16, 17, 22, 23, 29, 30 for C57BL/6 mice.

### Statistics

*In vitro* or *in vivo* data were expressed as the mean±s.e. of mean from indicated sample size. Differences between the means were analysed by one-way analysis of variance or Student's *t*-test. Statistical significance was considered when *P*<0.05.

## RESULTS

### Cyclosporin A enhanced Taxol-induced cytotoxicity in hepatoma cells

**Figure 1:**
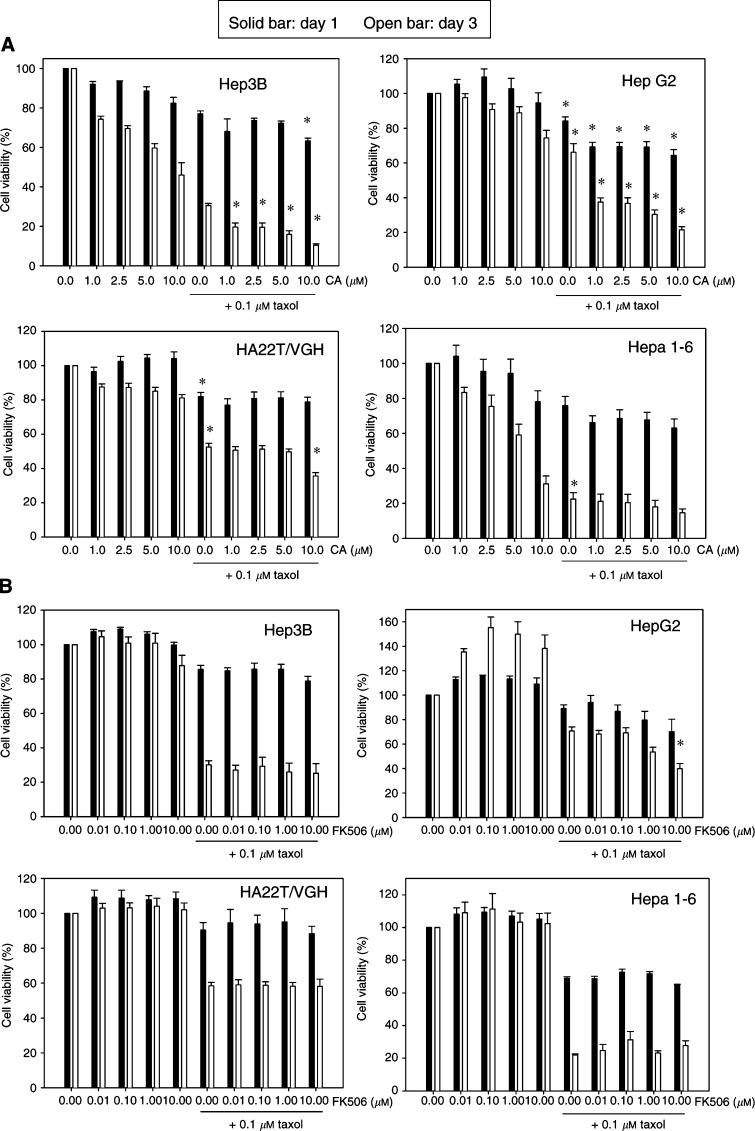
Effect of Taxol (0.1 *μ*M) and cyclosporin A (1–10 *μ*M) (**A**), or Taxol (0.1 *μ*M) and FK506 (0.01–10 *μ*M) (**B**) on cell viability of hepatoma cells. Data are expressed as mean±s.e. of mean from duplicate samples of three to five separate experiments. ^*^indicates *P*<0.05 as compared with respective control group.

We investigated the combination effect of cyclosporin A and Taxol on three human hepatoma cell lines (Hep3B, HepG2 and HA22T/VGH) and one murine hepatoma cell line (Hepa 1-6) (Figure 1A

). After cyclosporin A combined with Taxol treatment for 24 and 72 h, we found that cyclosporin A treatment alone resulted in decreased cell viability in Hep3B and Hepa 1-6 cells at 72 h after treatment. A dose-dependent decrease of cell viability was observed after cyclosporin A treatment. No significant decrease in cell viability was observed in HepG2 and HA22T/VGH cells after cyclosporin A treatment. These four hepatoma cell lines showed similar enhanced cytotoxic effect at 10 *μ*M cyclosporin A combined with 0.1 *μ*M Taxol treatment for 72 h. Therefore, we examined another immunosuppressant, FK 506, on the combination effect with Taxol. Figure 1B shows that no enhanced cytotoxic effect was observed in Hep3B, Hepa 1-6 and HA22T/VGH cells treated for 24 and 72 h. However, a partial effect was observed in HepG2 cells at 10 *μ*M of FK506 combined with 0.1 *μ*M Taxol treatment for 72 h.

### Drug-induced caspase activity

**Figure 2:**
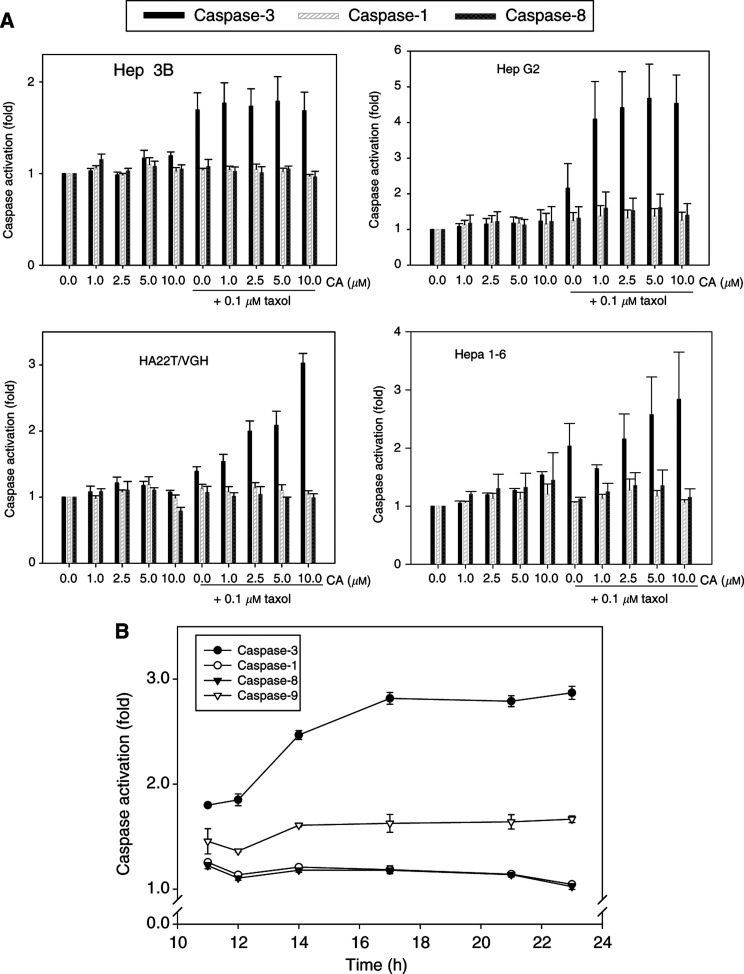
Effect of combined cyclosporin A (1–10 *μ*M) and Taxol (0.1 *μ*M) treatment on caspase-1, -3 and -8 activities in hepatoma cells at 24 h (**A**). The time-course response on caspase-1, -3, -8 and -9 activities after treatment of hepatoma cells with cyclosporin A (10 *μ*M) and Taxol (0.1 *μ*M) (**B**). Data are expressed as mean±s.e. of mean from duplicate samples of two to three separate experiments.

Either cyclosporin A or Taxol alone treatment had no significant effect on caspase-1, -3, and -8 activation in HepG2 and HA22T/VGH cells. In combination treatment of HepG2 cells for 24 h, the initially responsive dose of cyclosporin A was 1 *μ*M, which enhanced Taxol-induced caspase-3 activation, but not that of caspase-1 and -8. Identical results were observed at 10 *μ*M cyclosporin A combined with 0.1 *μ*M Taxol treatment in HA22T/VGH cells. For Hep3B and Hepa 1-6 cells, Taxol alone induced caspase-3 activation and no apparent augmentation was found after combination with cyclosporin A treatment (Figure 2A

). In order to determine the effect of cyclosporin A and Taxol combination treatment on the cascade of caspases activation, we investigated the membrane-derived signalling pathway, caspase-8 and mitochondria-derived signalling pathway, caspase-9, as well as caspase-1 and -3 activities in HepG2 cells. Figure 2B shows that activation of caspase-3 and -9 was observed within 11–24-h interval while no apparent changes was found between 1 and 10 h (data not shown). Moreover, caspase-1 and -8 were not significantly activated after combination treatment, indicating the specificity of caspase activation.

### Drug-induced loss of mitochondrial membrane potential

**Figure 3:**
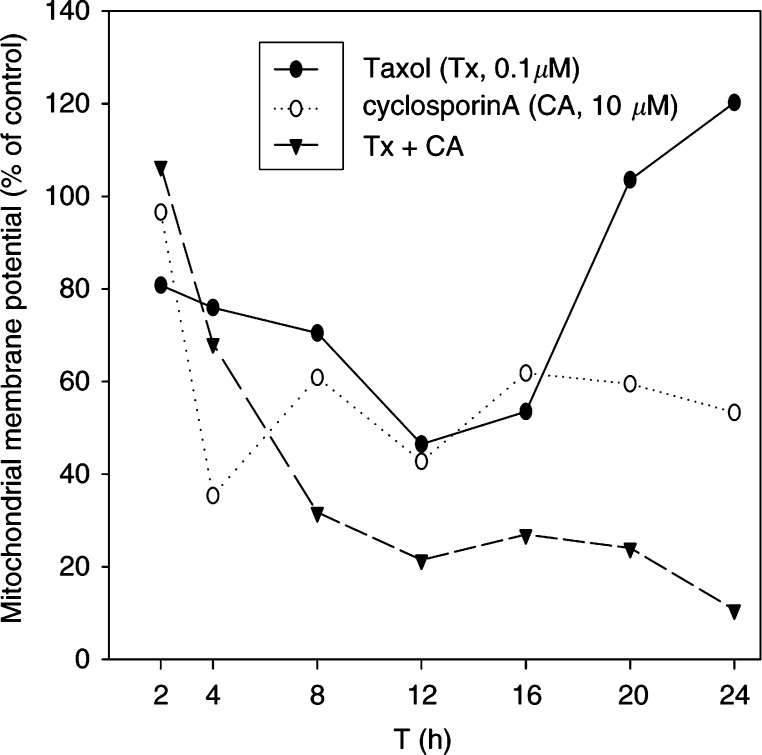
Percent changes in mitochondrial membrane potential after cyclosporin A (CA, 10 *μ*M) and Taxol (Tx, 0.1 *μ*M) treatment for 2–24 h in HepG2 cells.

Since initiator caspase-9 and executioner caspase-3 were activated after combination treatment, we further investigated whether caspase-9 activation was the result of mitochondria-mediated signals. It has been reported that loss of mitochondrial membrane potential elicited caspase-9-dependent apoptosis (Green and Reed, 1998). Thus, we utilised JC-1 as a specific probe for the determination of mitochondrial membrane potential. When the mitochondria maintain membrane potential, JC-1 (green fluorescence) forms the J-aggregates (red fluorescence). If mitochondrial membrane is depolarised, red fluorescence is reduced and eventually green fluorescence increased. As shown in Figure 3

, a combination treatment for 2–24 h enhanced the loss of mitochondrial membrane potential in HepG2 cells as compared to the Taxol or cyclosporin A alone treatment groups. In an attempt to analyse the time-course response of changes in mitochondrial membrane potential, we found that Taxol-induced a biphasic effect within 24 h. However, cyclosporin A and Taxol combination treatment led to inhibition of the Taxol-induced biphasic response and enhanced mitochondrial depolarisation.

### Effect of cyclosporin A and Taxol on signal transduction

**Figure 4:**
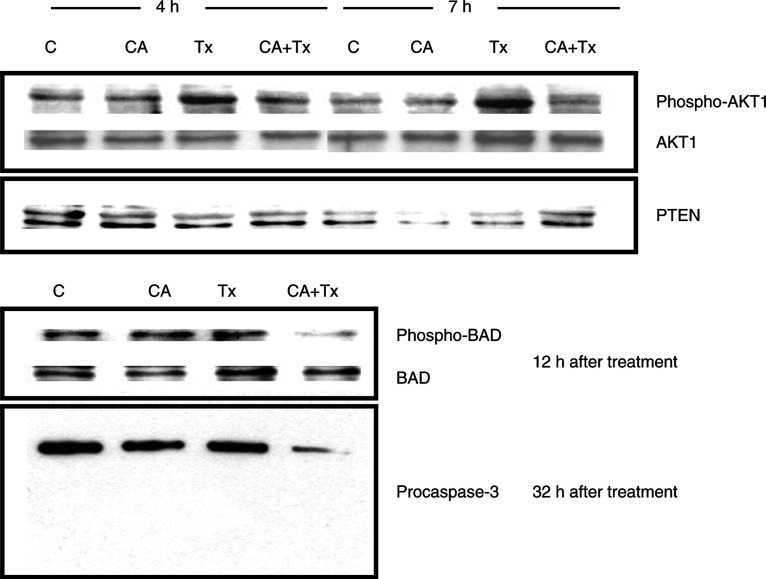
Western blot analysis of phospho-AKT1, AKT1, PTEN, phospho-BAD, BAD and procaspase-3 expression in HepG2 cells treated with cyclosporin A (CA, 10 *μ*M) and/or Taxol (Tx, 0.1 *μ*M).

Since the PI3-kinase-AKT pathway has been reported to be involved in cell survival response, we examined this pathway in cyclosporin A combined with Taxol treatment in HepG2 cells. Figure 4

shows that the level of phosphorylated active AKT1 (Ser 473) was increased after Taxol treatment and this increase was blocked by cyclosporin A combination treatment with Taxol (Figure 4). In addition, the AKT1 downstream protein, BAD, was phosphorylated by Taxol treatment and the phosphorylated level was reduced after combination treatment of Taxol and cyclosporin A. Next, we analysed the expression of a phosphatase, PTEN, which is reported to be responsible for the inactivation of AKT1 protein. We found that no change was observed at 4 h after drug treatment. However, PTEN expression was increased at 7 h after cyclosporin A and Taxol combination treatment. Moreover, we have found that the procaspase-3 level was reduced after combination treatment, which was consistent with the caspase-3 activation (Figure 2). In order to clarify the role of PI-3 kinase in Taxol-induced responses, we evaluated the cell viability by using a PI-3 kinase inhibitor, wortmannin (10 *μ*M), combined with Taxol. Table 1

**Table 1:** Cell viability of hepatoma cell lines after wortmannin (10 *μ*M) and Taxol (0.01 or 0.1 *μ*M) treatment for 72 h

	**Wortmannin (W)**	**Taxol (Tx)**	**W+Tx**
Hep3B^a^	78.02±1.06^b^	70.37±2.92	50.01±4.97
HA22T/VGH^a^	80.65±1.32	63.39±0.60	57.42±1.97
Hepa 1-6^a^	74.38±3.16	45.32±1.61	37.32±3.60
HepG2^a^	74.72±6.82	60.16±3.48	50.65±2.50
HepG2^c^	74.72±6.82	49.71±2.80	38.93±5.30

a The concentration of Taxol used in these cell lines was 0.01 *μ*M.

b Data are expressed as percentage of control.

c The concentration of Taxol used was 0.1 *μ*M.

shows that wortmannin enhanced the Taxol-induced cytotoxicity in four hepatoma cell lines. Further analysis of the caspase-3 activity showed that wortmannin-combined Taxol treatment significantly augmented caspase-3 activity as compared with either wortmannin or Taxol alone treatment (Figure 5A

**Figure 5:**
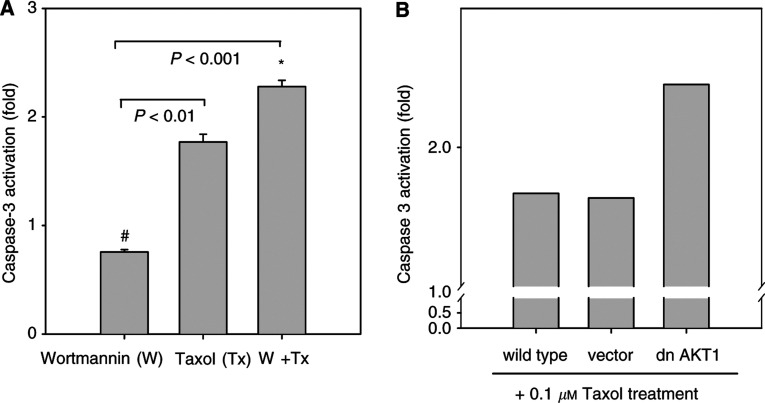
(**A**) Caspase-3 activity in HepG2 cells after wortmannin (10 *μ*M) and/or Taxol (0.1 *μ*M) treatment for 24 h. ^*^P<0.001 as compared with wortmannin alone treatment; #*P*<0.01 as compared with Taxol alone treatment, (**B**) Effect of a dominant-negative AKT1 expression vector (dn AKT) and control vector (vector) in combination with 0.1 *μ*M Taxol on caspase-3 activation. Caspase-3 activation is shown as fold of respective transient transfection control groups.

). To examine the role of AKT1 in Taxol-induced responses in HepG2 cells, a dominant-negative AKT1 expression vector was used. As shown in Figure 5B, transient transfection of a dominant-negative AKT1 in HepG2 cells had a similar effect on the enhancement of Taxol-induced caspase-3 activity by wortmannin.

### Tumour growth inhibition *in vivo*

**Figure 6:**
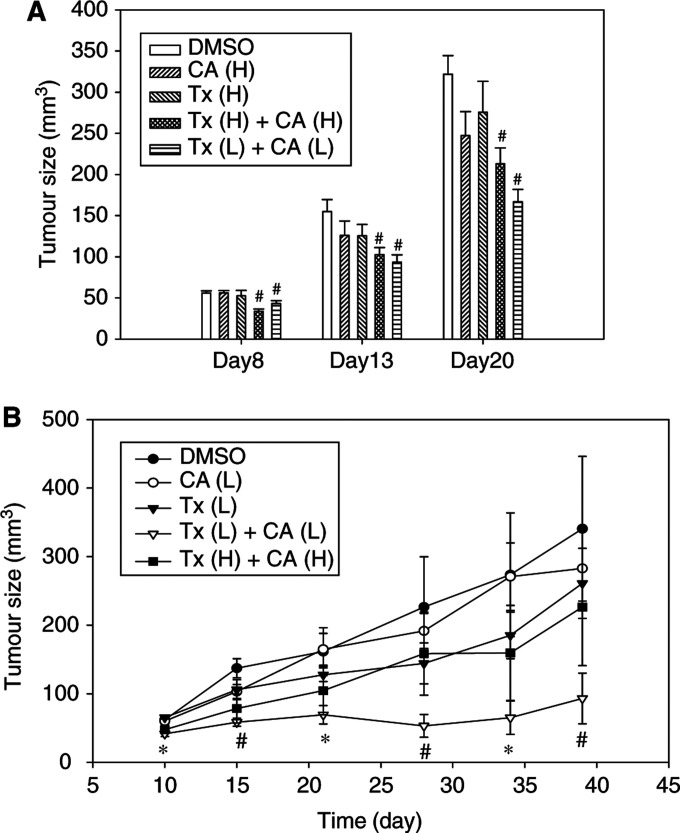
Growth inhibition of Hepa 1-6 tumours in SCID (**A**) and C57BL/6 (**B**) mice after treatment with cyclosporin A (CA) and/or Taxol (Tx). Cyclosporin A was given at two doses (CA (H): 6.67 mg kg^−1^; CA (L): 5 mg kg^−1^). The dose of Taxol was Tx (H): 2.68 mg kg^−1^ and Tx (L): 2 mg kg^−1^, respectively. Each point represents average from five to seven mice. The detailed procedures of animal studies were described in Materials and Methods. ^#^*P*<0.05; ^*^P<0.01 compared with DMSO control.

We evaluated the effect of cyclosporin A and Taxol combination treatment in SCID mice inoculated with Hepa 1-6 cells. To simulate the microenvironment of a solid tumour responding to chemotherapy, we injected Hepa 1-6 cells subcutaneously and produced a 0.2 × 0.2 cm^2^ (in diameter) small nodule in 3 days for the following studies. In a first set of experiments, SCID mice bearing Hepa 1-6 tumour were treated with combined cyclosporin A (6.67 or 5 mg kg^−1^) and/or Taxol (2.68 or 2 mg kg^−1^) twice a week for 3 weeks. Figure 6A

shows that cyclosporin A (6.67 mg kg^−1^) and Taxol (2.68 mg kg^−1^) treatment alone did not inhibit tumour growth. Similar results were observed with lower doses of cyclosporin A (5 mg kg^−1^) and Taxol (2 mg kg^−1^) (data not shown). In contrast, combination treatment resulted in significant growth inhibition of Hepa 1-6 tumour. The combination of a lower dose of cyclosporin A (5 mg kg^−1^) and Taxol (2 mg kg^−1^) showed a slightly better response than that of the higher dose of cyclosporin A (6.67 mg kg^−1^) and Taxol (2.68 mg kg^−1^) at 20 days after treatment. To confirm this observation, we performed a second experiment using an immune competent mouse C57BL/6 mouse model. Figure 6B shows that no significant growth inhibition of Hepa 1-6 tumour was observed in mice receiving either a lower dose of cyclosporin A (5 mg kg^−1^) or Taxol (2 mg kg^−1^). In contrast, significant tumour growth inhibition (*P*<0.05) was observed in mice receiving combination treatment. The growth inhibition was not the result of drug-induced toxicity because no loss of body weight was observed as compared with vehicle group (data not shown).

## DISCUSSION

Hepatocellular carcinoma is a solid tumour that is well known for poor response to chemotherapy. In a clinical trial using Taxol to treat 20 patients with HCC, no complete or partial response was observed (Chao *et al*, 1998). Frankel *et al* have indicated that cancer cells grown as spheroids showed decreased Taxol-induced cytotoxicity (Frankel *et al*, 1997). This corresponded well with our observation that no significant tumour regression was found after Taxol treatment (Figure 6). We have clearly shown that cyclosporin A and Taxol combination treatment resulted in the growth inhibition of Hepa 1-6 tumour cells not only in culture system (Figure 1) but also in an *in vivo* solid hepatoma model (Figure 6).

Clinically, venous invasion, multiple type and diffuse type of HCC limited surgical resection. Preoperative chemotherapy could provide an adjuvant therapy to facilitate reducing tumour size or number, and subsequently perform curative resection. Postoperatively, HCC recurrence in residual liver is a major problem in patients 1 year after surgical resection. It is estimated that 90% of HCC recurrence grows as multiple and nodule type, which not only decrease tumour response to chemotherapy but also limit repeated resection (Nagasue *et al*, 1993). Although Taxol-based therapy in patients with breast cancer showed efficient responses, Chao *et al* (1998) have found that Taxol failed to benefit patients with unresectable HCC. The observed poor response of these patients to Taxol corresponded well with our *in vitro* results, which showed that hepatoma cells had a different degree of resistance to Taxol (Lin *et al*, 1999,^2000^). These results further suggest that the Taxol resistance in HCC may involve poor uptake of Taxol because of the barrier in solid tumour as well as the intrinsic factors that regulate the metabolism of Taxol and cellular response to Taxol. The reversal of Taxol resistance in hepatoma cells suggests that combination of cyclosporin A and Taxol has potential for adjuvant therapy of HCC patients.

Identification of cellular targets of various drugs is important for the development of mechanism-based therapies. In this study, we have found that AKT1 was activated in Taxol-treated hepatoma cells. In addition, it was accompanied with phosphorylated downstream target protein – BAD (Figure 4). It has been reported that cytoplasmic phosphorylated BAD is involved in the prevention of mitochondria-derived apoptosis (Datta *et al*, 1997). Moreover, Cardone *et al* (1998) reported that AKT directly phosphorylated and inactivated caspase-9. Caspase-9 was found to be responsible for caspase-3 activation. Subsequent caspase-3 activation was required for a positive feedback loop in maintaining the status of loss of mitochondrial membrane potential (Heerdt *et al*, 1999). In this study, we have found that Taxol induced AKT1 activation in Taxol-resistant hepatoma cells (Figure 4). In contrast, AKT activated by HGF partially inhibited the Taxol-induced apoptosis in Taxol-responsive human U373 glioblastoma cells (Bowers *et al*, 2000). It is clear that initially Taxol induced loss of mitochondrial membrane potential (Figure 3) that was followed by AKT1 activation (Figure 4). Caspase-9 was possibly inhibited in part by AKT1 activation and blocked a positive feedback loop for sustained caspase-3 activation as well as sustaining loss of mitochondrial membrane potential. Both wortmannin and dominant-negative AKT1 expression vector partially reversed Taxol resistance in hepatoma cells (Figure 5), which further supports that AKT may coordinate the antiapoptotic activities and resulted in drug-resistant responses in hepatoma cells.

Another important intrinsic factor for Taxol resistance is the increased expression of MDR in hepatoma cells. Using rhodamine 123 fluorescence retention method (Lin *et al*, 1999) to evaluate the effect of cyclosporin A on the MDR1 transporter activity, we have found that cyclosporin A (1 *μ*M) had no effect on the function of MDR1 in HepG2 cells (data not shown). Therefore, the effect of cyclosporin A on the reversal of Taxol resistance in hepatoma cells most likely resulted from the modulation of the PI3 kinase-AKT1 signalling pathway rather than from the regulation of the MDR transporter system of hepatoma cells.

Finally, we conclude that cyclosporin A combined with Taxol treatment is effective against hepatoma growth *in vitro* and *in vivo*. The key to reversal of Taxol resistance may lie in the modulation of cellular signal transduction molecules such as AKT1 and PI3 kinase. Our results provided a mechanism-based research in combination treatment of HCC. Cyclosporin A and Taxol are clinically available drugs, the combination of these two drugs for pre- or postoperative adjuvant chemotherapy is worthy of clinical trial in patients with HCC.
